# Evaluating our ability to predict the structural disruption of RNA by SNPs

**DOI:** 10.1186/1471-2164-13-S4-S6

**Published:** 2012-06-18

**Authors:** Justin Ritz, Joshua S Martin, Alain Laederach

**Affiliations:** 1 Department of Biology, University of North Carolina, Chapel Hill, NC, 27599, USA

## Abstract

The structure of RiboNucleic Acid (RNA) has the potential to be altered by a Single Nucleotide Polymorphism (SNP). Disease-associated SNPs mapping to non-coding regions of the genome that are transcribed into RiboNucleic Acid (RNA) can potentially affect cellular regulation (and cause disease) by altering the structure of the transcript. We performed a large-scale meta-analysis of Selective 2'-Hydroxyl Acylation analyzed by Primer Extension (SHAPE) data, which probes the structure of RNA. We found that several single point mutations exist that significantly disrupt RNA secondary structure in the five transcripts we analyzed. Thus, every RNA that is transcribed has the potential to be a “RiboSNitch;” where a SNP causes a large conformational change that alters regulatory function. Predicting the SNPs that will have the largest effect on RNA structure remains a contemporary computational challenge. We therefore benchmarked the most popular RNA structure prediction algorithms for their ability to identify mutations that maximally affect structure. We also evaluated metrics for rank ordering the extent of the structural change. Although no single algorithm/metric combination dramatically outperformed the others, small differences in AUC (Area Under the Curve) values reveal that certain approaches do provide better agreement with experiment. The experimental data we analyzed nonetheless show that multiple single point mutations exist in all RNA transcripts that significantly disrupt structure in agreement with the predictions.

## Background

RNA (Ribonucleic Acid) is a ubiquitous messenger of genetic information in the cell and plays a central role in the regulation of molecular processes [1-5]. Unlike DNA, RNA is generally single stranded and has a high propensity to fold into functionally important structures [6-10]. These structures can be significantly disrupted by mutations including Single Nucleotide Polymorphisms (SNPs) [11,12]. Genome-Wide Association Studies (GWAS) regularly identify disease-associated SNPs in non-coding regions of the genome. Disease-associated SNPs do not necessarily directly reveal the molecular cause of the disease and require further analysis [11,13-15].

A majority of the genome is transcribed into RNA [16,17]; as a result a majority of genetic mutations will also be transferred to the transcriptome. From a structural perspective, we distinguish two broad classes of RNA; highly structured RNAs (e.g. the Ribosome, tRNAs, self splicing introns, RNAse P) and RNAs that potentially adopt multiple conformations (e.g. mRNAs and non-coding RNAs) [3,4,18]. Structured RNAs are under significant evolutionary pressure to adopt a single, functional conformation [19]. However, mRNAs and non-coding RNAs are not necessarily evolved to adopt a single conformation but rather adopt an ensemble of conformations [20-23]. We have recently found specific disease-associated mutations that alter the ensemble partitioning of mRNA affecting gene regulation and thus cause disease [24]. Thus, structure is likely an important functional feature even in RNAs traditionally thought of as “unstructured.”

Algorithms to evaluate the structural and functional consequences of mutations on proteins (e.g. PolyPhen and SIFT) are commonly used to assess the potential deleterious effects of mutations [25-27]. In addition, several groups are actively developing web servers to compute the potential deleterious effects of SNPs on RNA structure and function [28,29]. The structural basis for deleterious mutations to a structured protein is rationalized through an understanding of protein folding. For example, replacing a hydrophobic residue in the hydrophobic core of a protein with a hydrophobic amino acid will likely cause the protein to misfold [26,27]. In RNA however, the physico-chemical properties of the four-nucleotides are not as diverse as the amino acids. Furthermore, RNA does not fold through the formation of a hydrophobic core [4]. Instead the structure is a complex network of base-pairing and stacking interactions [3,8]. To observe a large conformational change in an RNA, the mutation must not only disrupt an existing base-pair, but also favor a completely alternative base-pairing network. The functional consequences of structure disruption depend on whether the affected region is involved in important regulatory interactions. In certain cases, small local changes in the RNA structure may have functional consequences [15,30]. In this manuscript we are interested in identifying the mutations that globally affect RNA structure and are thus likely to have significant functional consequences.

We initially interrogate high-throughput SHAPE chemical mapping of multiple non-coding RNAs and associated single point mutations [31,32]. We aim to determine whether single point mutations, like in proteins, can significantly alter the structure of the RNA. We then evaluate the performance of multiple RNA structure prediction algorithms to determine the optimal strategy for identifying the mutations that disrupt RNA structure. As GWAS (Genome Wide Association Studies) continue to focus more on non-coding regions of the genome, it will become increasingly important to have accurate algorithms for assessing the potential deleterious consequences of SNPs on the transcriptome.

## Results and discussion

### Single mutations disrupt RNA structure

**Figure 1:**
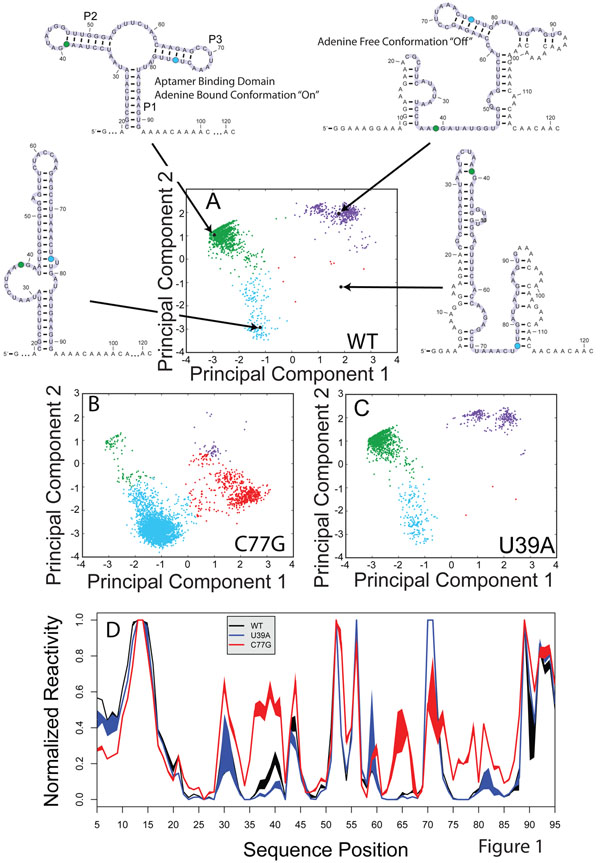
Structural analysis of the Adenine Riboswitch, which is a bacterial regulatory RNA that binds Adenine and controls gene expression [35,37]. The RNA adopts two major conformations, the “On” state (Adenine bound) forms three stem loops (P1, P2 and P3), while in the “off state” the site of translation initiation (3’ end of the UTR, near the start codon) is structured effectively disrupting translation initiation. A.) Boltzmann suboptimal sampling of the ensemble of possible RNA conformations (as predicted by sFold) projected onto the first two principal components of structure space as determined by a Manhattan distance metric evaluation of the ensemble. Each dot in the diagram is one alternative structure. Representative structures adorn the diagram, and the aptamer domain of the Riboswitch is highlighted in light magenta. The Riboswitch is predicted to adopt four structures, characterized by green, purple, cyan and red dots. The “on” and “off” states of the Riboswitch to the green and magenta cluters, respectively. B.) Boltzmann sampling of the structural ensemble for the C77G containing sequence which indicates a significant shift in partitioning towards the cyan and red conformations. C.) Boltzmann sampling for the U39A mutation which is predicted to have no effect on the partitioning compared to WT. D.) Experimental validation using SHAPE chemistry of the predictions made in A-C, showing that the C77G mutation disrupts the structure of the RNA in a manner consistent with an increase in the population of the cyan cluster.

To better understand the potential effects of SNPs on a large RNA we consider the Boltzmann sampled suboptimal ensemble of the *Vibrio vulnificus* Adenine Riboswitch (Figure 1A) [33,34]. Projecting these structures onto the first two principal components of their structural space as described previously [24], reveals four major clusters (Figure 1A). The Adenine Riboswitch is so named as the aptamer domain (highlighted in light magenta in Figure 1A) binds Adenine. It is one of the few Riboswitches that activates gene expression upon ligand binding [35-37].

The “on” and “off” conformations of the Riboswitch are present in the Boltzmann ensemble of the WT sequence (Figure 1A, green and magenta clusters, respectively). This is consistent with recent models that suggest that Adenine riboswitching is kinetically controlled at the transcriptional level [35]. Moreover, two other conformations (cyan and red clusters, Figure 1A) are not highly populated in the WT ensemble. If we repeat the Boltzmann sampling procedure for a sequence containing the C77G mutation (Figure 1B), we see a drastic shift in the ensemble favoring the cyan and red conformations. A majority of mutations, however, are like the U39A mutation and have very little effect on the suboptimal ensemble (Figure 1C).

To experimentally validate the prediction made by sub-optimal sampling made in Figures 1A-C, we queried the SNRNASM (Single Nucleotide Resolution Nucleic Acid Structure Mapping) archive as well as the RNA Mapping Database (RMDB, http://rmdb.stanford.edu) for chemical mapping data of the Adenine Riboswitch [38]. We found SHAPE chemical mapping data for the WT, C77G and U39A transcripts under identical solution conditions (10 mM MgCl_2_ and 100 mM KCl). This data provides single nucleotide resolution measurements of base-pairing in the Riboswitch [39]. A high normalized SHAPE reactivity indicates high flexibility and thus low probability of base-pairing, while low reactivity indicates high likelihood of base-pairing [40,41]. The data in Figure 1D therefore experimentally validates the predictions made in Figures 1A-C. We see that the C77G (red trace) is significantly different from the black (WT) and blue (U39A) traces, consistent with a large shift in the predominant structures in the ensemble. The significant increase in SHAPE reactivity in residues 32-43 and 62-68 are consistent with the hairpin structure represented by cyan cluster.

We compute the experimental Structure Disruption Coefficient (eSDC) to evaluate the effect of a SNP on the RNA structure as described in the Methods (Equation 1). This value measures the disruptive effect of a SNP on an RNA, the higher it is the greater the structural disruption. In this case it is 2.0 for C77G and 0.1 for U39A. Furthermore, we can use the multiple repeats of the experiments to evaluate the statistical significance (p-value) of these eSDC values, i.e. the probability that we would obtain the value due to noise in the data. For the C77G, the difference is statistically significant (p-value < 0.001) while for U39A it is not (p-value >0.5).

### Systematic eSDC analysis of five non-coding RNAs

**Figure 2:**
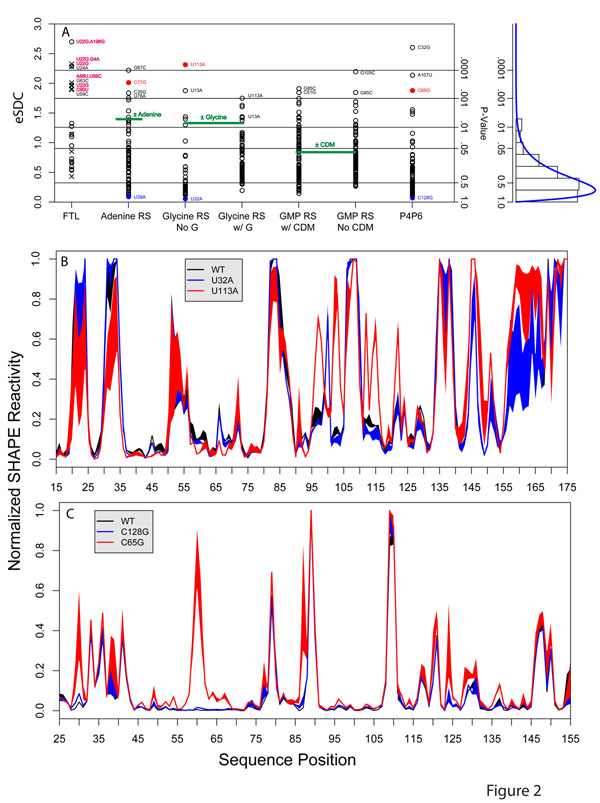
Comprehensive analysis of mutation induced structure disruption in five non-coding RNAs. A.) eSDC (experimental Structure Disruption Coefficient) for 470 single or double mutatants relative to the RNA’s WT sequence. eSDC is computed as one minus the Pearson correlation coefficient of the SHAPE profile (mutant to WT) multiplied by the square root of the length of the RNA. We see that most mutations have small eSDC values indicating that they do not significantly disrupt structure. The five RNAs studied are the human FTL 5’ UTR (FTL), the *V. vulnificus* Adenine Riboswitch (Adenine RS), the *V. cholera* Glycine Riboswitch (Glycine RS with and without Glycine (G) bound), the cyclic di-GMP Riboswitch (bis-(3'-5')-cyclic dimeric guanosine monophosphate Riboswitch with and without cyclic-diguanosine-monophosphate (CDM)) and the P4P6 domain of the L-21 *Tetrahymena thermopila* group I intron [5,34,35,63]. All data were collected under near physiological solution conditions, i.e. 10mM MgCl_2_ and 100 mM monovalent. For FTL, hyperferritinemia associated mutations are indicated in magenta. The eSDC values for ± ligand for the three Riboswitches are indicated with a green horizontal line and represent a “biological” threshold above which a structure change is likely to have a functional consequence. This histogram to the right represents a pairwise “within” eSDC calculation for 6-fold repeats of the SHAPE experiments on the FTL UTR RNA to evaluate the reproducibility and significance (p-value) of eSDC values. B.) SHAPE profiles for the WT, U32A and U113A (black, blue, and red respectively) Glycine Riboswitch in the presence of Glycine showing that the U113A mutation very significantly disrupts structure. C.) SHAPE profiles for WT, C128G and C65G (black, blue, and red respectively) P4P6 group I intron transcripts showing that the C65G globally affects the structure of the RNA.

The SNRNASM and RMDB databases contain 470 SHAPE data sets of RNA sequences with single and/or double point mutations relative to WT RNA for five non-coding RNAs under similar monovalent and divalent salt concentrations. We therefore computed eSDC values for these 470 mutations and summarize the results in Figure 2A. In all cases we computed eSDC values relative to the WT sequence to identify single or double mutations that significantly disrupt RNA structure.

The results of our analyses are plotted on Figure 2A and reveal that in all cases certain mutations (e.g. U22G.A196G in FTL, U113A in the Glycine Riboswitch) significantly disrupt RNA structure. However, a majority of mutations (e.g. U39A and U32A in the Adenine and Glycine Riboswitches) have very small effects on structure. We plotted representative SHAPE data for structurally disruptive (red) and non-disrupting mutations for the Glycine Riboswitch and P4P6 intron in Figures 2B and C, respectively. To evaluate the significance of the structural disruption, we computed the “within” distribution for multiple repeats (6-fold) of the FTL UTR RNA SHAPE data and plot the resulting histogram to the right of Figure 2A. This allows us to determine the expected eSDC values due to the noise in the experimental data, and evaluate the p-value for any given eSDC. Clearly, single point mutations exist that significantly disrupt RNA structure, however a majority of mutations result in no measurable effect.

The FTL UTR data set is particularly interesting, as this RNA is a “RiboSNitch,” i.e. an RNA in which specific SNPs can alter structure and cause disease [24,42]. In this case, FTL is associated with Hyperferritinemia Cataract Syndrome, a rare genetic disorder that is characterized by early onset cataracts due to excess ferritin in the retina [43,44]. We indicate the disease-associated SNPs as magenta text in Figure 2A. All the disease-associated SNPs alter the structure of the RNA significantly (p-value < 0.001).

Three of the RNAs tested in Figure 2A are Riboswitches and undergo a conformational change if ligand is present. We can therefore compute an eSDC value for SHAPE traces in the presence and absence of ligand. We indicate these eSDC values with a green horizontal line in Figure 2A. The reason this result is important is that the structural change caused by ligand binding to a Riboswitch is sufficient to regulate gene expression [37,45,46]. Thus the Riboswitch ligand eSDC value (green line Figure 2A) represents a “biological” threshold above which the structure change is likely to affect function. A particularly important result of this analysis is the identification of multiple SNPs with much larger eSDC values compared to ligand binding in the Riboswitches. Thus, it is likely that a majority of these SNPs will have important functional consequences.

### Performance of RNA structure prediction algorithms for RiboSNitch detection

**Figure 3:**
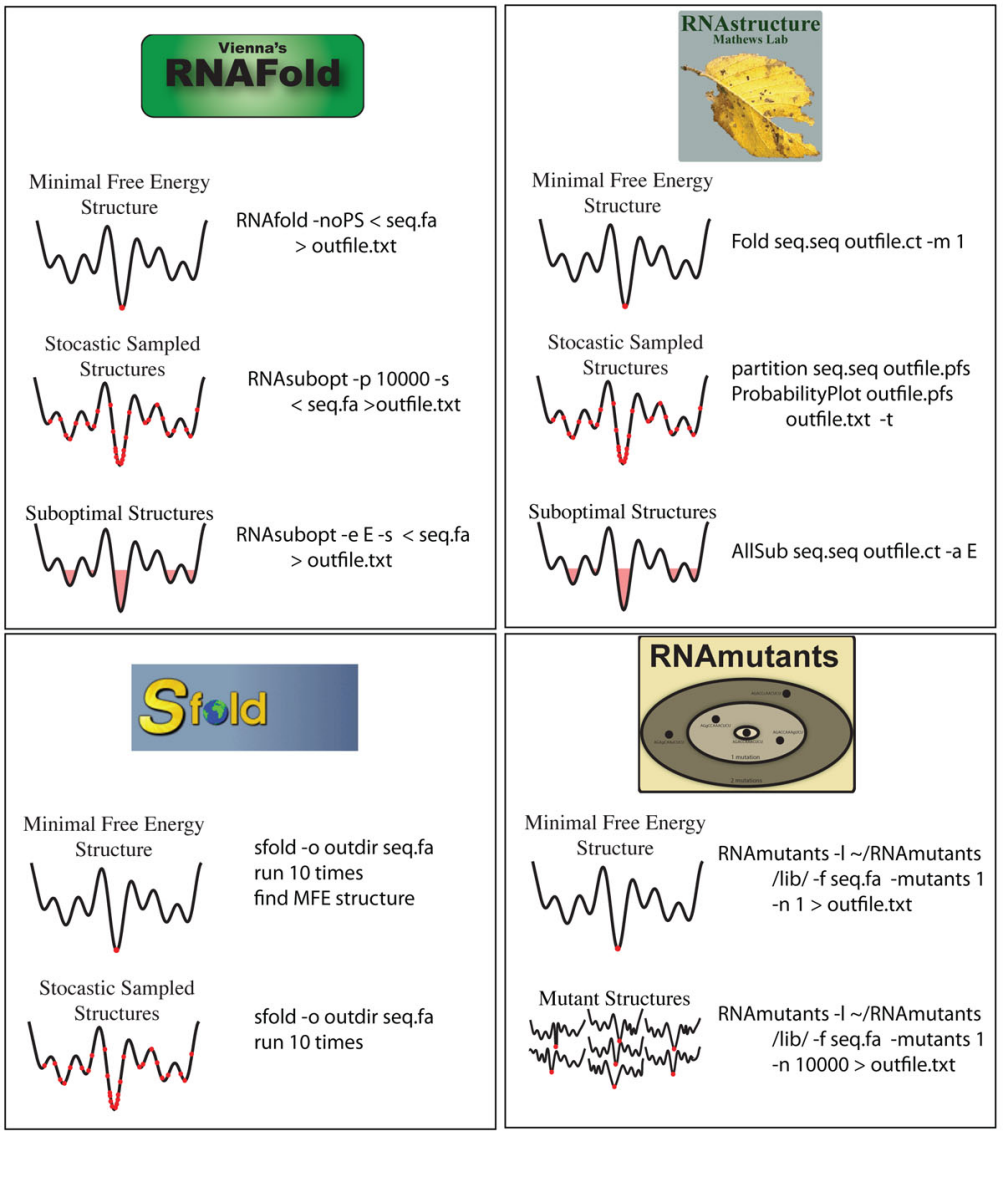
Schematic representation of the four software packages we benchmarked for their ability to predict which mutations in an RNA affect structure most significantly. We chose these packages as they all perform some form of sub-optimal sampling, illustrated with “cartoon” energy landscapes. We also include the precise UNIX commands used to make the predictions.

We chose to benchmark the four software packages illustrated in Figure 3 [23,47-49], as they each have various options to evaluate the ensemble of suboptimal structures. The precise UNIX commands we used to generate the predictions are also indicated in Figure 3. It should also be noted that all of these programs are designed to predict the best secondary structure, and with the exception of RNAmutants are not necessarily optimized for identifying the mutation that most disrupts RNA structure.

**Figure 4:**
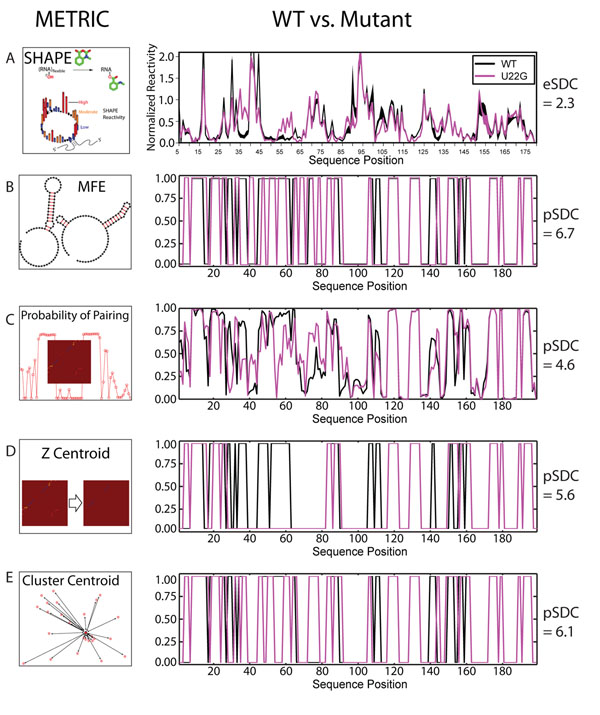
Schematic representation of metrics used to compute pSDC (predicted Structural Disruption Coefficients) based on RNA structure predictions for WT (black) and mutant (magenta). The data here are for the WT, and hyperferritinemia cataract syndrome associated U22G mutant of the FTL 5’ UTR. A.) SHAPE experimental data for the WT and U22G mutant UTRs revealing a significant effect of the U22G mutation on the structure of the RNA. An eSDC value of 2.3 is computed for this data. B.) sFold Minimum Free Energy (MFE) probability of base-pairing for the WT (black) and U22G (magenta) containing sequence, one corresponds to not-base-paired and zero paired. We see that the program correctly predicts changes in the 40-60 range as measured by SHAPE. C.) Probability of base-pairing computed as the sum of the rows or columns of the partition function [64]. In this case the partition function is computed using sFold Boltzmann suboptimal sampling and computing the observed frequency of base-pairing [51]. D.) Z Centroid simplification of the partition function and probability of pairing computed by summing the rows or column [51]. E.) Probability of pairing assessed as the cluster centroid structure of the most populated cluster of suboptimal structures, in this case using sFold and k-means clustering as previously described [51].

We aim to use RNA structure prediction programs to predict the eSDC values determined from the SHAPE data (Figure 4A). Figure 4 illustrates the four metrics applied to the ensemble of structures from each algorithm and used to generate pSDC values (predicted Structure Disruption Coefficients, Equation 4, methods). This metric is analogous to the eSDC as it allows us to rank order SNPs according to their predicted disruption of RNA structure. All structure prediction programs we tested can compute a Minimum Free Energy (MFE) structure. We represent this as a vector of ones and zeroes, and compute the correlation coefficient between the WT and mutant structures (Figure 4B). Many structure prediction algorithms can also compute the probability of base-pairing (which is more analogous to SHAPE reactivity) by summing the rows or columns of the predicted partition function matrix (Figure 4C) [48,50]. We computed the Z Centroid (Figure 4D) of the partition function as well [51]. Finally, for the algorithms that sample suboptimal structures, we can cluster the resulting ensemble and determine the centroid structure for the most populated cluster (Figure 4E) [23,51].

**Figure 5:**
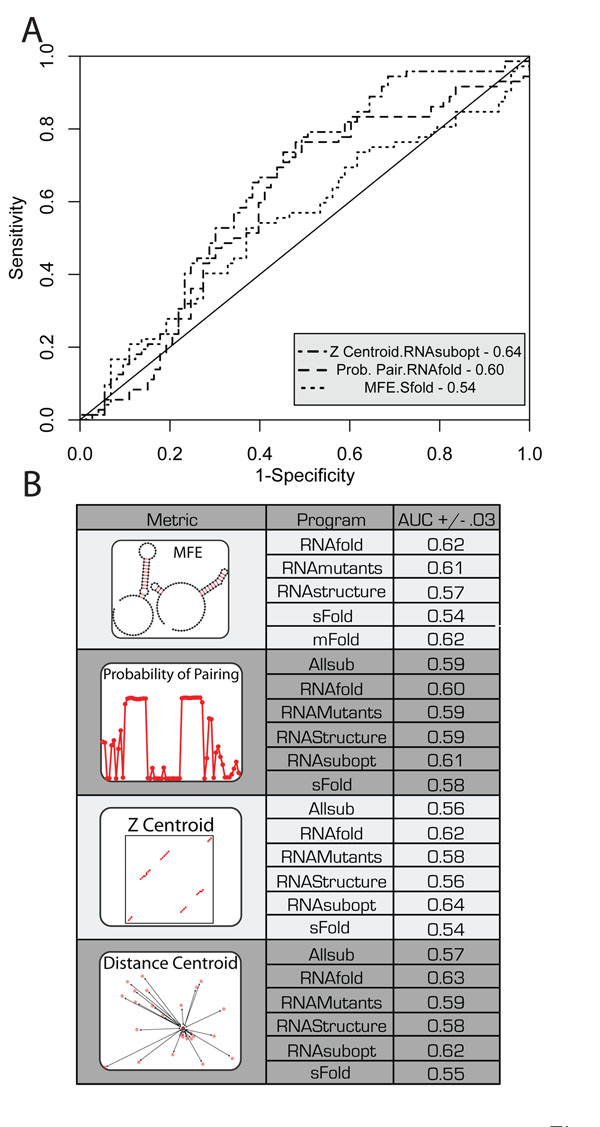
Evaluation of the different pSDC metrics and RNA structure prediction algorithm’s performances. A.) Receiver Operator Characteristic (ROC) analysis of three representative metric/algorithm combinations indicating significant differences in the predictive performance. The three curves are for the RNAsubopt prediction of the Z centroid structure (for the first 10,000 structures), base-pair probability as computed from the partition function of RNAfold, and the MFE structures predicted by sFold. B.) Summary of all Area Under the Curve values for ROC analysis of metric/algorithm combinations. In general, most algorithms perform equivalently for identifying the mutations that disrupt RNA structure.

We found that in general pSDC values are larger than eSDC values. We are most interested in the different algorithms’ (Figure 3) and metrics’ (Figure 4) ability to rank and identify the mutations that maximally disrupt structure. To evaluate each algorithm’s performance we generated Receiver Operator Characteristic (ROC) curves based on the ranking of the 470 mutant RNA’s eSDC values (Figure 2A) compared with those ranked by pSDC. Figure 5A plots three representative ROC curves and illustrates that algorithm/SDC metric combinations vary in their predictive performance. The AUC (Area Under the Curve) values reported in Figure 5B suggest that the highest performing algorithm is RNAsubopt using a Z centroid metric (AUC 0.64). The “partition function” for RNAsubopt was obtained by computing the pair probabilities for the first 10,000 suboptimal structures. The AUC values reported in Figure 5B reveal that most algorithm/metric combinations perform similarly and are within the standard error of 0.03 when the experimental data is bootstrapped. eSDC values, and SHAPE data for all mutants analyzed are provided as tables in the additional files. Additional Files 1-8 correspond to the FTL 199, FTL 226, Adenine RS, Glycine RS NoGlyc, Glycine RS wGlyc, GMP RS wCDM, GMP RS NoCDM, and P4P6, respectively.

## Conclusions

RNA is a ubiquitous regulatory molecule in the cell and there is growing evidence that structure is a central component of its function [52,53]. The Riboswitches studied in this manuscript are one of many examples where RNA structure change regulates bacterial metabolism [46,54,55]. In the case of the 5’ UTR, disease-associated SNPs disrupt structure and deregulate Ferritin levels in the eye, resulting in early onset cataracts [24]. The *T. thermophila* group I intron (P4P6) must fold into its correct three-dimensional structure to self-catalyze its splicing reaction [8,56]. In these examples, structure change is central to the RNA’s function in the cell.

The data we present in Figures 1 and 2 reveals the extent to which a single point mutation can disrupt RNA structure. Our systematic analysis of 470 mutations on five RNAs reveals that large scale SNP induced structure change is common in RNA and can potentially contribute to disease [24]. Interestingly, all RNA secondary structure prediction algorithms predict that a small subset of mutations will have a large effect on secondary structure. The data we present in Figures 1 and 2 cover a relatively comprehensive set of mutations in each RNA, but are nonetheless limited to five functional molecules. As such, the generalizability of these results will require the analysis of larger experimental data sets as they become available [38].

The mechanism for this change is best illustrated in Figure 1, where we see how a single mutation (in this case C77G) can completely alter the thermodynamic folding landscape of the RNA, favoring an alternative conformation. The data we present in Figure 2 suggest that the thermodynamic models used to predict RNA structure are sound, as we find mutations experimentally in all RNAs studied that disrupt structure. All RNA structure prediction algorithms predict that certain mutations will significantly disrupt structure. In addition, a recent study of common SNPs in the human genome revealed that these affect local RNA structure [57].

An important result in our analysis of the Riboswitch SHAPE data is the comparison of the eSDC values for mutations relative to ligand induced conformational change (see green lines, Figure 2A). For all three Riboswitches, multiple mutations exist that result in far larger structural changes (as measured by our SDC metric) than ligand binding. This is highly relevant, as ligand binding induced structure change can completely turn on (or off) gene expression translationally and/or transcriptionally [45]. Thus the mutations above the green lines in Figure 2A have even greater potential to regulate cellular function. This means any functional RNA has the potential to be a “RiboSNitch,” as there exists mutations that can significantly disrupt its structure.

The data we present in Figure 2 are ideal for benchmarking RNA structure prediction algorithms. The analysis we carried out in this manuscript is different from previous secondary structure prediction benchmarks, because we are specifically interested in identifying mutations that globally disrupt a given secondary structure. We developed metrics based on RNA secondary structure prediction algorithms analogous to our eSDC calculations. We can use such an analogy, since SHAPE data is correlated with base-pair probability. The SDC metrics are purposefully global, and we did not evaluate algorithms for their ability to predict the specific local changes in structure, but rather whether they predict that a specific mutation will disrupt structure relative to others. Our reasoning for this approach is that for the analysis of disease-associated SNPs, we are most interested in identifying the most structurally deleterious mutations.

Although RNA structure prediction algorithms correctly predicted that all RNAs are disrupted by certain mutations, it is clear that predicting exactly which mutation will alter structure remains very challenging. Although there is some variation in the relative performance of the different algorithmic and metric combinations we tested, the AUC values reported in Figure 5B remain relatively low. This result is not necessarily surprising, as none of the RNA structure prediction algorithms (other than RNAmutants) have been optimized to predict which mutations disrupt structure. In fact, an algorithm’s sensitivity to point mutations is often viewed as a weakness, favoring methods that are less sensitive to mutation. However, the experimental data clearly show that SNPs can profoundly change an RNA’s folding landscape.

The attempts to constantly refine algorithms so as to have them always converge on a single “correct” RNA structure may not improve their ability to identify RiboSNitches. Although only anecdotal, mFold’s good performance in our benchmark (AUC 0.62, Figure 5B) may indicate that simpler energy functions, which tend to predict more alternative structures, may ultimately perform better for identifying RiboSNitches. Indeed RNAStructure’s relatively low performance in our benchmark is surprising, since it has the most sophisticated and accurate energy function and is most accurate in structure prediction [48,50]. Improvements in our ability to predict RiboSNitches will likely require a better understanding of the suboptimal ensemble and how mutations affect it in addition to improved energy functions. With the growing number of sequencing efforts revealing ever more single nucleotide variants in the non-coding regions of the genome, accurate algorithms predicting the structural consequences of these mutations are likely to play an important role in genomic interpretation.

## Methods

### Data collection and analysis

The SHAPE chemical data used in our analysis were downloaded in ISATAB format from the SNRNASM (Single Nucleotide Resolution Nucleic Acid Structure Mapping) and RMDB web sites (http://snrnasm.bio.unc.edu and http://rmdb.stanford.edu). The SNRNASM standard was developed to share the results of high-resolution and throughput nucleic acid structure mapping data [58]. We identified RNAs that were probed using SHAPE chemical mapping under standard conditions (10 mM MgCl_2_ and 100 mM NaCl), and where significant mutational information was available. Only RNAs that were at most two SNPs (or mutations) away from a reference (WT) sequence were considered. The data were normalized as previously described [59], and for the two Riboswitch and P4P6 data sets, manually re-aligned to correct for frameshift errors due to the automated analysis of the data using the HiTRACE software [42]. eSDC values were computed as described by Equation 1:(1)

where *^p^CC* is the WT/mutant pearson correlation coefficient and *n* is the length of the RNA. The eSDC quantitatively evaluates the effect of a mutation on RNA structure. Prior to the calculation of the eSDC, normalized SHAPE values were capped at one in order to increase the metric's ability to reflect changes in structure identified by differences in the peaking pattern and not minor differences in peak intensity. Significance testing for structure disruption was adjusted using a Bonferroni correction.

### PCA analysis of the ensemble of structures and clustering

Principal components were calculated (as described previously) from a total of 10,000 sampled structures generated equally from a WT sequence and mutants of interest [24]. The principal components were generated from the binary representation of these 10,000 structures. These structures were then projected onto the first two principal components and subjected to the k-means clustering algorithm to reveal distinct clusters [60]. The centroid structure of each cluster was identified from the k-means clustering algorithm and then drawn using R2R [61]. Individual mutant structures were then generated (as discussed in Fig. 3) and projected onto the first two principal components. Each structure projection is colored according to their cluster.

### Computation of the partition functions from sampled structures and calculation of the Z centroid

Partition functions were generated for each ensemble of structures. Each structure is first transformed to matrix form as described in [51]. This is accomplished by creating an NxN matrix where N is the length of the sequence and placing a 1 at position i,j and j,i if nucleotides i and j are paired and a 0 if they are not paired. When all the matrices representing the structures are summed together and then divided by the total number of structures, the resulting matrix is the partition matrix. This matrix contains the probability of nucleotide i being paired to j. The Z centroid is defined as the structure with all the probability of pairing for each pair greater than 50%.

### ROC analysis of prediction performance

Each of the program/metric combinations were evaluated using a Receiver Operator Characteristic (ROC) Analysis [62]. The ROC analysis was carried out by calculating the true positive rate (*i.e. sensitivity*):(2)

and false positive rate (*i.e. 1-specificity*):(3)

from the True Positives (TP), False Positives (FP), True Negatives (TN) and False Negatives (FN):(4)

Analogously to the *eSDC* calculation, we compute a *pSDC* (predicted Structure Disruption Coefficient) by computing the Pearson Correlation Coefficient (*^pred^CC*) between WT and mutant for each RNA structure prediction algorithm. This value is analogous to the eSDC in that it allows us to rank order the disruptive effect of mutations on RNA.

To determine ROC values, the mutations were listed from highest to lowest according to their eSDC value. The top 50% of eSDC values were considered to disrupt the structure while the lowest 50% preserved structure. A second list was generated using the same mutants but using the pSDC values instead. A true positive was defined as having a pSDC value above a cutoff and experimentally disrupting the structure while a true negative was defined as having a pSDC below a cutoff and experimentally preserving a structure. A false positive or false negative is recorded when the predictions contrast with the experimental results. The pSDC cutoff was defined by stepping through the pSDC ranks. The resulting true positive rates and false positive rates were then used to generate an ROC curve. The area under the curve was calculated for each ROC using the trapezoidal method. This process was bootstrapped for each program/metric 5000 times using 20 randomly selected mutants from each set. Due to the fact that each of the RNA data sets has a differing number of mutants, the bootstrapping is done by sampling 20 mutants from each of the other data sets besides FTL, in order to correct for any bias that might come up due to one program/metric favoring one data set over another. This results in the ROC being run on 145 mutants at a time, not the full 470. The average area under the curve was calculated with the standard deviation between runs generating the error. The closer the area under the curve was to one the better the predictive power for a given program/metric.

Precise WT sequences, corresponding mutations (SNPs), eSDC values and normalized SHAPE data are provided as separate excel spreadsheets in the additional files. These data should facilitate further benchmarking efforts for novel algorithms to predict RNA structure change.

## List of abbreviations

eSDC: experimental Structure Disruption Coefficientl; pSDC: predicted Structure Disruption Coefficient; SHAPE: Selective 2'-Hydroxyl Acylation analyzed by Primer Extension; SNP: Single Nucleotide Polymorphism; FPR: False Positive Rate; TPR: True Positive Rate; SNRNASM: Single Nucleotide Resolution Nucleic Acid Structure Mapping.

## Competing interests

The authors declare that they have no competing interests.

## Author’s contributions

JR performed the analysis of the SHAPE data, determined statistical significance and conceived the statistical tests. JM performed the structural predictions, made Figures 3 and 4. AL directed the research and wrote the manuscript.

## Supplementary Material

Additional file 1 eSDC and SHAPE data for the FTL 199 nucleotide length RNA construct. Click here for file

Additional file 2 eSDC and SHAPE data for the FTL 226 nucleotide length RNA construct. Click here for file

Additional file 3 eSDC and SHAPE data for the Adenine Riboswitch RNA construct. Click here for file

Additional file 4 eSDC and SHAPE data for Glycine Riboswitch without Glycine RNA construct. Click here for file

Additional file 5 eSDC and SHAPE data for the Glycine Riboswitch with Glycine RNA construct. Click here for file

Additional file 6 eSDC and SHAPE data for the GMP Riboswitch with CDM RNA construct. Click here for file

Additional file 7 eSDC and SHAPE data for the GMP Riboswitch without CDM RNA construct. Click here for file

Additional file 8 eSDC and SHAPE data for the P4P6 subdomain of the *Tetrahymena thermophila* group I intron RNA construct. Click here for file
